# P02.66. Efficacy of acupuncture for fibromyalgia - RCT

**DOI:** 10.1186/1472-6882-12-S1-P122

**Published:** 2012-06-12

**Authors:** S Vinjamury, J Jones, L Hsiao, M Moutappa, J Weiss

**Affiliations:** 1 Southern California University of Health Sciences, Whittier, USA; 2 California State University, Fullerton, Fullerton, USA

## Purpose

The purpose of this study was to compare the efficacy of acupuncture and simulated acupuncture in patients with fibromyalgia.

## Methods

Fifty fibromyalgia patients were randomized into two groups based on predetermined eligibility criteria. Experienced acupuncturists provided real or simulated acupuncture two to three times per week to complete ten sessions within four to six weeks. Fibromyalgia Impact Questionnaire (FIQ), Multidimensional Pain Inventory (MPI), Composite Physical Function Scale (CPF) and 30-second chair stand were used to determine the improvements in pain, physical function, and lower body strength, respectively. Data were collected at baseline, at the end of the fifth treatment, at the end of the tenth treatment and at six-month follow up. The study was approved by the local IRB.

## Results

Thirty-nine participants completed the study. Demographic and disease characteristics were similar at baseline in both groups. Preliminary results indicate no significant main effects for group x time interactions (between groups). However, significant overall changes were seen within groups on outcome variables across time. No serious adverse events were reported by the participants. The dropouts were equal between the two groups. Blinding was effective.

## Conclusion

A fixed point acupuncture protocol as adopted in this study was no better than simulated acupuncture in relieving pain or improving overall functionality in fibromyalgia patients. The study is limited by the small sample size. Further trials that adopt individualized treatments are recommended with larger sample sizes.

